# Protocol to evaluate the effectiveness and cost-effectiveness of an environmental nutrition and physical activity intervention in nurseries (Nutrition and Physical Activity Self Assessment for Child Care - NAP SACC UK): a multicentre cluster randomised controlled trial

**DOI:** 10.1186/s12889-023-16229-y

**Published:** 2023-08-02

**Authors:** Ruth Kipping, Miranda Pallan, Kim Hannam, Kate Willis, Alex Dobell, Chris Metcalfe, Russell Jago, Laura Johnson, Rebecca Langford, Corby K. Martin, William Hollingworth, Madeleine Cochrane, James White, Pete Blair, Zoi Toumpakari, Jodi Taylor, Dianne Ward, Laurence Moore, Tom Reid, Megan Pardoe, Liping Wen, Marie Murphy, Anne Martin, Stephanie Chambers, Sharon Anne Simpson

**Affiliations:** 1 University of Bristol, Bristol, UK; 2 University of Birmingham, Birmingham, UK; 3 NatCen Social Research, London, UK; 4 Pennington Biomedical Research Center, Baton Rouge, USA; 5 Cardiff University, Cardiff, Wales; 6 Bristol Trials Centre, University of Bristol, Bristol, UK; 7 University of North Carolina, Chapel Hill, USA; 8 MRC / CSO Social & Public Health Sciences Unit, University of Glasgow, Glasgow, UK; 9 School of Social and Political Sciences and MRC/CSO Social and Public Health Sciences Unit, University of Glasgow, Glasgow, UK

**Keywords:** Obesity prevention, Child, Early years, Nursery, Physical activity, Nutrition, Prevention

## Abstract

**Background:**

One in seven UK children have obesity when starting school, with higher prevalence associated with deprivation. Most pre-school children do not meet UK recommendations for physical activity and nutrition. Formal childcare settings provide opportunities to deliver interventions to improve nutritional quality and physical activity to the majority of 3–4-year-olds. The nutrition and physical activity self-assessment for childcare (NAP SACC) intervention has demonstrated effectiveness in the USA with high acceptability in the UK. The study aims to evaluate the effectiveness and cost-effectiveness of the NAP SACC UK intervention to increase physical activity, reduce sedentary time and improve nutritional intake.

**Methods:**

Multi-centre cluster RCT with process and economic evaluation. Participants are children aged 2 years or over, attending UK early years settings (nurseries) for ≥ 12 h/week or ≥ 15 h/week during term time and their parents, and staff at participating nurseries. The 12-month intervention involves nursery managers working with a Partner (public health practitioner) to self-assess policies and practices relating to physical activity and nutrition; nursery staff attending one physical activity and one nutrition training workshop and setting goals to be achieved within 6 months. The Partner provides support and reviews progress. Nursery staff receive a further workshop and new goals are set, with Partner support for a further 6 months. The comparator is usual practice. Up to 56 nurseries will be stratified by area and randomly allocated to intervention or comparator arm with minimisation of differences in level of deprivation. Primary outcomes: accelerometer-assessed mean total activity time on nursery days and average total energy (kcal) intake per eating occasion of lunch and morning/afternoon snacks consumed within nurseries. Secondary outcomes: accelerometer-assessed mean daily minutes of moderate-to-vigorous physical activity and sedentary time per nursery day, total physical activity on nursery days compared to non-nursery days, average serving size of lunch and morning/afternoon snacks in nursery per day, average percentage of core and non-core food in lunch and morning/afternoon snacks, zBMI, proportion of children who are overweight/obese and child quality-of-life. A process evaluation will examine fidelity, acceptability, sustainability and context. An economic evaluation will compare costs and consequences from the perspective of the local government, nursery and parents.

**Trial registration:**

ISRCTN33134697, 31/10/2019.

## Background

Childhood obesity is one of the major public health challenges of the 21st century and has been exacerbated by the COVID-19 pandemic. In England and Scotland around 14% of children [1, 2] are living with obesity when they start primary school (age 4–5 years), with the highest rates of obesity in the most deprived areas. This is an increase of nearly 5% from pre-pandemic levels [1]. Consequently, there is a need for effective obesity prevention approaches to target the pre-school age group, that can be delivered at scale.

Diet and physical activity (PA) are key behavioural targets for obesity prevention interventions. PA in childhood is associated with lower levels of cardio-metabolic risk factors and improved psychological well-being [3], and PA intervention studies in young children have consistently reported improved motor and cognitive development, and psychosocial and cardiometabolic health [4]. PA behaviours track from childhood into adulthood, thus positive patterns should be established in the early years of life [5]. The UK Chief Medical Officer recommends that children aged 1–5 years should be physically active for at least 3 h per day, with 1 h of this being moderate-to-vigorous PA (MVPA) [6]. In 2015, fewer than 9% of 2–4 year olds in England were meeting the PA guidelines and 83% of children had ‘low’ activity levels [7]. This signals a need for improvement in children’s PA levels.

The amount of PA in which a young child engages is influenced by the activity undertaken at pre-school and particularly the time spent outside [8]. A UK study reported that children aged 3 to 4 years, spend more time in MVPA and are less sedentary in pre-school and nursery settings compared with time spent at home [9]. However, the time spent in MVPA is still low, with one UK study reporting only 9% of children’s time at preschool being spent in MVPA [10]. The lack of MVPA in pre-school settings may be influenced by space and equipment, policies (including scheduled times for free/outdoor play), and staff training in PA promotion [11].

Dietary patterns established during childhood influence those in later life [12, 13]. Obesogenic dietary patterns, characterized by low intake of fruits, vegetables, high-fibre breakfast cereals (core foods) [14] and high intake of chocolate, confectionery, low-fibre bread, biscuits and cakes (non-core foods), have been observed in young children and are associated with a higher risk of adiposity in later childhood [15, 16]. Food provided in early years settings potentially contributes to these obesogenic dietary patterns, with non-core foods contributing to around 40% of energy intake in younger children (aged 1–3 years) when in attendance at these settings [17]. In terms of nutrient intake, only 15% of younger children meet the UK recommendation of free sugars contributing to no more than 5% of total energy intake, and this falls to 2% in the 4–10-year age-group. For fibre intake, only 12% of the younger children and 14% of the older children meet the recommended intake [18]. In addition to diet quality, meal size is a critical driver of weight gain. In children aged 2–5 years, every extra 10 kcal consumed per meal are associated with a 7% faster rate of weight gain [19]. A survey 10 years ago of early years settings in the UK found those in the most deprived areas reported serving more healthy food (whole grains, legumes, pulses, and lentils), however many were not meeting national guidelines [20]. There are no data on portion size of servings to children in early years settings in the UK.

Early-years settings provide scalable opportunities to deliver diet and PA interventions at the population level [21] and are considered important environments for early intervention to establish positive health behaviours and prevent childhood obesity [22]. In England and Scotland, 94% and 97% of 3–4 year-old children respectively, access the Government-funded early-years education [23, 24] and a large proportion of children under 5-years also attend private- or voluntary-funded childcare organisations (nurseries) beyond this funded provision [25], presenting an opportunity to positively influence eating and PA. However, currently there is limited guidance for PA and nutrition in early-years settings. UK voluntary guidelines exist for food in these settings [21, 26], but in contrast to school settings, where national school food standards legislation [27] applies, there are no statutory standards for food provision. Similarly, schools have a statutory requirement to provide physical education [28], but there are no PA-related requirements in early years settings.

### Physical activity and nutrition interventions in early years settings

Several syntheses of obesity prevention, PA, and nutrition intervention studies in children under 5 years of age provide some evidence of the effectiveness of these interventions, including those delivered in early years settings [29–33]. These evidence syntheses have identified a clear need for more robust research in this area, including evaluation of interventions with explicit theoretical underpinning and the inclusion of economic evaluation. A systematic review by Larson et al. [30] explored policies, practices and interventions for promoting healthy eating and PA in childcare settings and concluded that there was little focus on promoting these behaviours. They emphasised the opportunity for interventions and policies to support healthy eating and PA in these settings.

The 2019 Cochrane review of interventions for preventing obesity in children [34], which included 22 studies of interventions in childcare settings, highlighted the need for a better understanding of intervention implementation and collection of data to allow exploration of the impact of intervention on health inequalities. The latter is particularly important as obesity prevalence in UK children is more than double in deprived areas compared with the most affluent areas [1]. This also signals the need for early-years settings interventions to be designed to particularly benefit low-income families and other groups with poorer nutrition, lower physical activity and higher obesity prevalence.

In the UK, there have been few randomised-controlled trials (RCT) of early-years setting interventions targeting diet, physical activity and obesity [35–37], with mixed findings. One cluster-RCT of an early-years educational intervention showed a small reduction in BMI standard deviation score (zBMI) in the intervention, compared with the control group [35]. Another cluster-RCT evaluating a PA intervention in early-years settings reported no difference in physical activity or BMI between intervention and control groups; the authors suggested the intervention was probably of inadequate dose [37].

### The nutrition and physical activity self assessment for child care (NAP SACC) programme

The NAP SACC programme [38] was developed in the USA and is an early-years setting intervention which aims to improve policies, practices, and the nutrition and PA environment, through a process of self-assessment and targeted assistance. NAP SACC is informed by social cognitive theory (SCT), which identifies the interrelationship between the environment, people, and behaviour [39], within a socio-ecological framework, which identifies multiple, interdependent influences at policy, community, organisational, interpersonal, and intrapersonal levels [40, 41]. The goals of the NAP SACC programme are to improve the nutritional quality of food served, the amount and quality of PA, staff-child interactions, and nutrition and PA policies [38].

Several RCTs of the NAP SACC programme in the USA have demonstrated its feasibility, acceptability and effectiveness, reporting: improvements in environmental audit nutrition scores [42]; increases in staff knowledge of childhood obesity, healthy eating, personal health, and working with families; decreases in children’s zBMI (-0.14; 95% CI -0.26,-0.02) [43]; and increased accelerometer-measured PA by 17% [44], following delivery of the NAP SACC programme. No studies included an economic evaluation or assessed dietary intake as an outcome.

### Feasibility of the NAP SACC UK intervention

In partnership with stakeholders, the NAP SACC programme was adapted for use in the UK (NAP SACC UK, details of which are published elsewhere [45]) and a feasibility cluster-RCT conducted with 168 children aged 2–4 years in 12 nurseries in North Somerset and Gloucestershire, England in 2015 to 2016 [45–47]. Overall, the NAP SACC UK intervention was delivered as planned, except for the home component (designed to support parents with their child’s PA and nutrition which was found not to be feasible), and the trial methods and design were found to be acceptable and feasible.

Post intervention (8–10 months after baseline), total activity in nursery settings was higher in the intervention nurseries compared with the control nurseries by 18.7 min/day (95% CI 3.8, 41.3) using the (underpowered) complete-case multi-level linear regression model adjusted for baseline outcome, age, gender and average hours of attendance. Evidence was less clear for improvements in anthropometry and dietary practices in the nurseries; however, the nursery managers reported improvements in several areas of feeding practice.

Given these promising outcomes of the NAP SACC UK feasibility study, together with the limited UK-based evidence for early-years settings interventions targeting diet and physical activity (particularly those targeting the early-years settings environment), there is a need to conduct a more definitive evaluation of the NAP SACC UK intervention. In response to the feasibility study findings, the intervention has been further refined by adapting the timings of intervention processes, extending the intervention period to 1 year (to include two Review and Reflect and goal setting cycles and a top-up day workshop), including lunchboxes in the Review and Reflect and workshop content and allowing for flexibility around the types of Local Authority staff who can deliver the intervention. Additionally, to improve dietary assessment, detailed data on food served and consumed using food photography will be captured to improve the assessment of diet. This will provide evidence for effectiveness and cost-effectiveness of an intervention that targets the eating and PA environments of early-years settings, which could potentially be rolled out at scale in the UK.

## Methods/design

### Study aims and objectives

The aim of the trial is to evaluate the effectiveness and cost-effectiveness of the NAP SACC UK intervention to increase physical activity and diet quality, while reducing sedentary time and portion size to nationally recommended levels, using a cluster RCT design with embedded process and economic evaluations. The trial will take place in early years settings (referred to throughout as “nurseries”). The co-primary objectives are to determine whether the NAP SACC UK intervention at 12 months:

- increases mean accelerometer-measured total physical activity on nursery days compared with usual practice
- reduces the energy (kcal) per eating occasion averaged across snack and lunch eating occasions that occur within nurseries compared with usual practice, within Nationally recommended levels.

The secondary objectives are to determine whether the NAP SACC UK intervention, compared with usual practice at 12 months:

- increases the mean moderate to vigorous physical activity time per nursery day
- reduces the mean sedentary time per nursery day
- increases the difference in mean accelerometer-measured total physical activity on nursery days compared to non-nursery days
- reduces the mean serving size of lunch and morning/afternoon snacks in nursery per day
- increases the balance of grams of core food to grams of non-core food consumed for lunch and morning/afternoon snacks in nursery per day
- reduces child zBMI
- reduces the proportion of children with overweight/obesity
- improves child quality of life
- is cost-effective
- is delivered with fidelity and in a way which is acceptable and sustainable.

### Study design

**Fig. 1:**
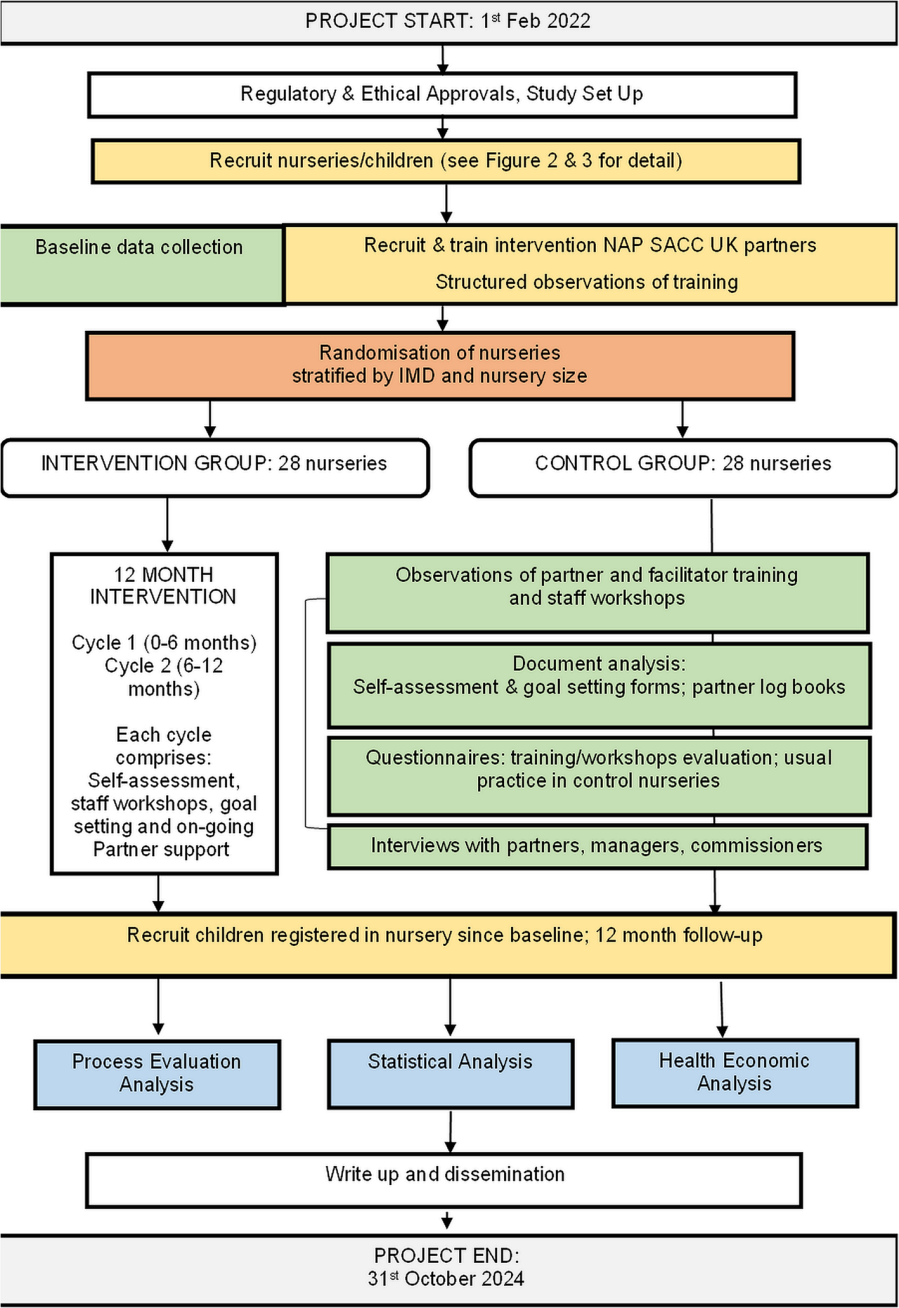
NAPSACC UK overview flow chart

**Fig. 2:**
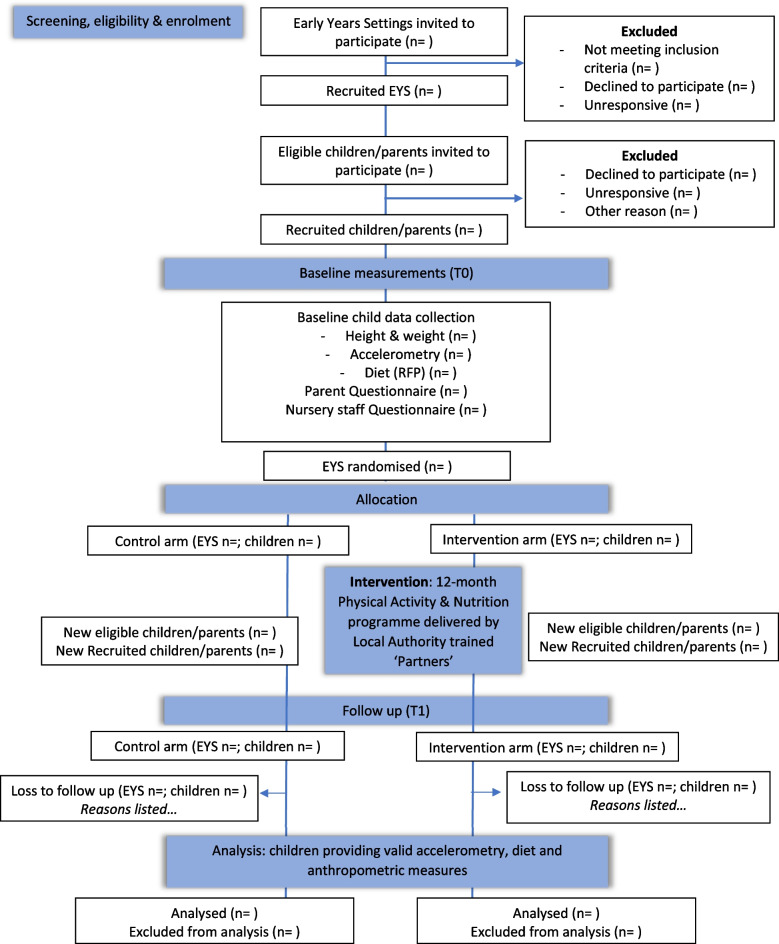
SPIRIT flow diagram for NAPSACC UK study

The NAP SACC UK study is a multicentre, parallel-group, two-arm, cluster RCT with a repeat cross-sectional design. Clusters (nurseries) will be randomised to receive either the 1-year NAP SACC UK intervention or continue with usual practice. The effectiveness and cost-effectiveness of NAP SACC UK will be assessed immediately after the 1-year intervention. Figure 1 provides a study overview and Fig. 2 provides a SPIRIT flow diagram outlining the stages of the study.

Separate cross-sectional samples of children attending the participating nurseries will be taken at two time points. The first will be prior to randomisation (T0) and the second, immediately after the intervention (T1). NAP SACC UK is designed to be an environmental intervention influencing the whole nursery which will impact all children and not just those present at T0. Due to the movement of children during the intervention period, the children at T0 may not be representative of the cluster, thus the repeat cross-sectional design may minimise bias and has been used in previous studies [48].

### Setting and participants

The trial will include up to 56 nurseries from four areas across England and Scotland: Ayrshire and Arran (Scotland), Sandwell, Somerset and Swindon (England). These areas were selected to ensure a broad range of deprivation status and ethnicity (non-white population varying from 2% in Somerset to 30% in Sandwell [UK 2011 census]) to enable exploration of generalisability. The research will be managed from three University ‘Hubs’ local to the study areas (University of Bristol, University of Glasgow and University of Birmingham).

Nurseries within the four study areas are eligible to participate if they are: day nurseries, private nursery schools, maintained nurseries (including nurseries within Children’s Centres), nursery classes attached to primary schools and pre-schools where children consume lunch (provided by the nursery or family). Nurseries will be excluded from participating if they are: childminders, crèches, playgroups, primary school reception classes, solely outdoor nursery settings, solely Special Educational Needs and Disabilities (SEND) nursery settings, au pairs, or settings taking part in a research study or other initiative that would interfere with the NAP SACC UK study.

Participants within each nursery setting include nursery staff (managers and childcare staff), parent/carers and children. Child (and associated parent/carer participants) inclusion criteria are: aged 2 years or over at the time of assessment, not yet attending Reception (England) or Primary One (Scotland), attending the participating nurseries for a minimum of 12 h/week across the year or 15 h/week during term time, and consuming at least lunch within the setting.

### Recruitment

#### Recruitment of nurseries

The study aims to recruit nurseries across the Index of Multiple Deprivation (IMD) or Scottish Index of Multiple Deprivation (SIMD). Within each of the four sites (S)IMD scores will be assigned to all nurseries and invitations sent across the range. An email will be sent to potentially eligible settings which will include a short informative film about the study, study summary and participant information sheet. The email will be followed up by a telephone call from the research staff to the nursery manager to undertake a screening check for eligibility and offer a meeting to discuss further study details. If eligible nursery managers decide to participate in the study, they will be provided with a consent form and letter of agreement to sign. Up to 56 nurseries will be recruited with the aim of a balance across the four sites. All nursery staff from recruited settings who work directly with children aged 2 years and over will be invited to participate.

#### Recruitment of parent/carers and children

In selected and consented nurseries, all parent/carers of children aged 2-years or over will be informed about the study and invited to participate if their child attends for the minimum eligible hours. Parents will have the opportunity to review study documents as hard copies or online and view a short informative online film. Opt-in consent will be obtained for parents as participants, as well as on behalf of their associated child. The research team will be available by email or telephone to answer questions and, if appropriate, in person at a convenient time for the nursery.

### Random allocation

Each nursery will be randomly allocated to the NAP SACC UK intervention or usual practice comparator group once all T0 data have been collected from the children, parents and staff at the nursery. Allocation will be conducted by a statistician, blind to the identity of nurseries and otherwise uninvolved in the ongoing study.

Within each hub separately, the allocation of nurseries will be conducted to minimise differences on an average IMD score (created for each nursery using the postcodes of the children recruited) at each site. Each random allocation will attempt to balance the IMD score between the two groups per site. This minimisation procedure will be written in Stata and code included in the Statistical Analysis Plan.

### Blinding

Two statisticians and two health economists will support this trial. The senior statistician and health economist will be blinded throughout the trial and will not have access to any identifying data. A study statistician and a health economist will perform all disaggregated analyses according to a pre-specified statistical analysis plan and health economic analysis plan, respectively. In addition, the study statistician will attend Trial Steering Committee meetings as required and prepare all interim reports, e.g., on recruitment and data completeness. The remaining members of the study team will remain blinded to aggregate data only.

### Sample size

Our aim is to recruit up to 56 nurseries (784 children), allowing for two nurseries withdrawing from the study and an average of 14 children per nursery, allowing for up to 35% failing to provide valid accelerometer data on nursery days. Assuming nine children will provide valid primary outcome data at each nursery, 27 nurseries in each of the intervention and control arms will provide 90% power at the 5% significance level to detect a 17-min difference (0.4 standard deviations) in total daily physical activity on nursery days. In the absence of a good estimate of the variation in mean total activity per day between nurseries, we allowed for variation up to a magnitude corresponding to an intra-cluster correlation of 0.087. The coefficient of variation of cluster size is 0.3 to account for slightly variable cluster size, i.e., different numbers of children in nurseries. As our measure of nutrition is on a continuous scale, a trial of 56 nurseries will be able to detect a 0.4 standard deviation difference in kcal, under the same assumptions. From our feasibility data, this is about 45 kcal which equates to approximately half a banana or half a cup of milk.

### Comparator group provision

All consenting nurseries, nursery staff, children and parents/carers will participate in the baseline (T0) and follow-up (T1) data collection. However, the control nurseries and participants within them, will not receive the NAP SACC UK intervention and will continue practice as usual. Data will not be collected from control nurseries for the economic analysis.

### Data collection

Table 1 outlines the data collection time points at the nursery, child and parent level. Details of data collection methods for PA, diet and anthropometric data are also listed here.

**Table 1:** NAPSACC UK SPIRIT schedule of enrolment, interventions and data collection

	**Screening eligibility check**	**Consent**	**Baseline data (T0)**	**Intervention/ Usual Care**	**Screening eligibility check for new children registered with nursery**	**Consent for new children registered with nursery**	**Follow-up data (T1)**
**Nursery**							
Demographics	•						
Consent		•					
Number of eligible children		•					
Staff mediator questionnaire			•				•
Observation of training^a^				•			
Staff questionnaires after workshops^a^				•			
Review and reflect tool^a^				•			
Goal-setting forms^a^				•			
Nursery staff time and NAP SACC partners time and costs^a^				•			^b^
Staff interviews							•
**Child**							
Demographics	•				•		
Consent		•				•	
Height			•				•
Weight			•				•
Accelerometer activity			•				•
Diet (RFPM)			•				•
Child Quality of Life (PedsQL)			•				•
**Parent**							
Demographics	•				•	•	
Consent		•			•	•	
Parent mediator questionnaire			•				•
Data linkage consent to school height and weight			•				•

^a^Intervention nurseries only

^b^The time taken to complete each intervention component (e.g. workshops, ‘review and reflect’ consultations and ongoing technical assistance) will be electronically logged by the Partner immediately after each activity for the full 12-month intervention period

#### Physical activity

Accelerometers will be worn for five week days.

#### Diet data

Morning/afternoon snack and lunch diet data will be collected in nursery from consented children using the Remote Food Photography Method (RFPM) to give direct estimation of portion sizes of foods and drinks through visual comparison of photographs to standard portion size photographs [49, 50]. A researcher will take one photograph of a child’s eating occasion before consumption and one photo after. Photos are taken at a 45-degree angle and approximately at arm’s-length distance from the plate, including all the food on the plate. If a child receives additional portions of food, a photograph will be taken of the plate before the serving is added and again after to capture a photograph of the new portion. A final photo is then taken when the child finishes eating to capture any leftovers or confirm the entire serving was consumed. The photographs will be annotated to assist with food identification or portion size estimation where relevant and submitted for analysis to Pennington Biomedical Research Centre (PBRC) [51]. The photos will be processed to estimate nutrient composition of foods consumed. In the absence of the meal being weighed or specific recipe being provided from the nursery, standard recipes or volumetric portion sizes will be used based on the National Diet and Nutrition Survey (NDNS) nutrient databank [52].

#### Anthropometric measures

All anthropometric measurements will be completed with children in a private area with a member of nursery staff. Weight will be measured without shoes in light clothing to the nearest 0.1 kg using a calibrated medical grade digital scale. Height will be measured to the nearest 0.1 cm, without shoes, using a portable stadiometer. Fieldworkers will be trained to ensure correct position for height assessment.

### Process evaluation

Two elements have been identified as critical to successful implementation: 1) the valued relationship formed between the nursery manager and NAP SACC UK Partner and 2) the motivation and “buy in” created among nursery staff at the workshops [47]. Local Authorities (or the NHS Board in Scotland) have identified relevant health or health improvement staff to take on the roles of Partners to replicate what is likely to happen in any future implementation. However, each group of staff will be trained to the same specifications. The process evaluation will explore this variation to understand its impact on how the intervention was implemented and received. The process evaluation will explore the following components:

- *Fidelity*: the degree to which the intervention happened as planned in each area?
- *Acceptability*: acceptance of the intervention to nursery managers, Partners and Local Authority/NHS Board commissioners.
- *Sustainability*: exploration of the appropriateness of differing models of Partner provisions; if [and how] NAP SACC UK becomes embedded into nursery and Partner processes.
- *Context*: the way in which context affected the fidelity, acceptability and sustainability of the intervention and how it interacted with the hypothesized mechanisms of the intervention.

The process evaluation will use a combination of methods to collect detailed information to contextualise the results of the trial and inform any potential roll-out plans should the intervention prove effective:

- Semi-structured observations of Partner training and staff workshops, to explore fidelity and quality of implementation
- Questionnaires to capture i) staff views on the quality and utility of the training workshops and ii) usual practice in control nurseries.
- Semi-structured interviews with NAP SACC Partners and intervention nursery managers to explore implementation, acceptability, sustainability and control. Local commissioners in each area will also be interviewed to explore sustainability and fit with local priorities.
- Document analysis of self-assessment and goal-setting forms to assess changes made because of the intervention.

### Economic evaluation

Data will be collected to capture the costs and consequences from the perspective of the local government, nurseries and parents. The primary economic outcome will be child health-related quality of life, measured at baseline and 12 month follow-up using the parent-reported PedsQL for 2–4 year olds. The developer of the PedsQL measurement tool advised that this measure would be suitable for parents in our study who are completing the measure for a 5 year old child. Versions of the PedsQL for older children include questions about school which would not be relevant to our participants. We will capture costs related to each perspective using various methods, including:

- *Intervention training costs (local government perspective):* Study records will document all costs incurred during the 2-day Partner training to deliver the NAP SACC UK intervention.
- *Intervention delivery costs (local government perspective):* NAP SACC UK Partners will complete an electronic log where they will record the contact time they had with each nursery provider. The Partners will also be asked to record details on the type of nursery staff whom they have been in contact with and the mode of delivery.
- *Implementation costs (nursery perspective):* At the 6-month point in the delivery of the intervention - all nursery managers for the intervention arm will be asked to complete a short health economic questionnaire. To prepare the nursery managers, they will be sent the questionnaire in advance to report on whether any of the actions they undertook as part of the NAP SACC UK intervention, have had an impact on staff time and/or resulted in a financial cost to the nursery during the previous 6-month period. Nursery managers will also be asked whether the workshops took place out-of-hours and if this incurred a financial cost to the nursery. At 12 months, a subsample (~ 50%) of nursery managers from the intervention group will be asked to complete the same health economic questionnaire. In addition, the subsample of nursery managers who are taking part in the process evaluation will also be invited to repeat the health economic questionnaire at the 12-month time point.
- *Participation costs (parent perspective):* At 12 months, time and out-of-pocket costs incurred by the parents during the 12-month intervention period will be captured through parental self-report questionnaires.

Our feasibility study indicated insufficient value in collecting information from parents on their children’s use of healthcare during the intervention to justify the burden. Healthcare use in this generally healthy population was infrequent and believed to be very unlikely to be causally related to the intervention.

### Participant appreciation

To thank all participating nursery schools, in both the intervention and control arms, we will provide £300 on completion of the study, along with a summary of results at nursery level. Following data collection, participating children will receive a small token of thanks in the form of a sticker and a children’s book; parents will receive a £10 voucher on return of their child’s accelerometer.

### Intervention

Table 2 outlines the detail of the intervention using the TIDieR reporting guidance as a framework. Local Authorities have chosen the most appropriate locally employed staff to deliver the intervention (public health practitioners with expertise in public health, nutrition or physical activity who are referred to as NAP SACC UK Partners), to enable us to test the effectiveness of the intervention as it might be delivered outside a trial.

**Table 2:** NAPSACC UK TIDieR framework outlining the intervention details

Item	Description
Name	Nutrition and Physical Activity Self-Assessment for Child Care UK (NAP SACC UK)
Why	NAP SACC UK is an intervention delivered in childcare settings with the aim of improving the nutrition and physical activity environment, through a process of self-assessment and targeted assistance. NAP SACC UK is a theory-based programme that employs components of social cognitive theory (SCT) and the socio-ecological framework. The objectives of the programme are to improve the nutritional quality, variety and quantity of food served, amount and quality of physical activity, staff-child interactions and staff behaviours around nutrition and physical activity and childcare provider policies.
What: materials	The NAP SACC UK intervention is based around a self-assessment tool (the ‘Review & Reflect’) completed by nursery managers with advice and support from a NAP SACC UK Partner. This is a 100-item multiple choice questionnaire, completed by the nursery manager, covering areas in nutrition, physical activity and play, outdoor play and learning, and screen time. Following completion of the ‘Review & Reflect’, the nursery manager along with the NAP SACC UK Partner agree on eight goals: three nutrition, three physical activity and a further two of the nursery’s choice.
What: procedures	The NAP SACC UK intervention is a five-stage process: 1. Self-Assessment. 2. Workshop delivery: Specialised staff deliver workshops to all nursery staff on: i) Nutrition; ii) Physical Activity. 3. Goal setting and Action Planning: The NAP SACC UK Partner works with the nursery manager to develop an action plan, listing eight goals for improvement. 4. Tailored technical assistance: NAP SACC UK Partner continues regular contact with nursery to provide support and advice toward them meeting their goals. 5. Evaluate, revise, repeat. The ‘Review & Reflect’ self-assessment is repeated by the nursery manager after 6 months and reviewed with the NAP SACC UK Partner to see where improvements have been made or not, and to explore ways to overcome barriers; action plans are revised to set eight new goals for the next 6 months.
Who provided	Within each local authority, NAP SACC UK Partners who deliver the nursery workshops will be chosen from a range of health or health improvement staff with appropriate skills. All staff will be provided with 1 day of training led by specialists in nutrition and physical activity who provided the training in the feasibility study.
How	The main part of the intervention will be delivered face to face; this includes NAP SACC UK Partners going through the ‘Review & Reflect’, action planning and delivering the workshops. Other parts of the intervention, such as on-going support and advice from the NAP SACC UK Partner can be provided over the phone, by email or face to face. All parts of the intervention will be delivered to participating nurseries individually. Some parts may be delivered on a one-to-one basis (e.g. nursery manager and NAP SACC UK Partner setting goals), while other parts such as the workshops will be delivered to a group of staff from one nursery. NAP SACC UK Partners will have 4 days contact with each nursery over the 12 months.
Where	The NAP SACC UK intervention is delivered in the nursery itself. The NAP SACC UK Partner offers visits to the nursery and the workshops take place at the nursery.
When and how much	The NAP SACC UK intervention takes place over 12 months. The length of the workshops are a total of 6 h, followed by a 2 h workshop after 6 months. The nurseries receive ongoing regular support over the 12 months.
Tailoring	The technical assistance offered by the NAP SACC UK Partner will depend on the goals.
Modifications	In the feasibility study the intervention was 5 months; in the full trial it will be 12 months. NAP SACC was designed in the US to be for a year and this longer period enables a mid-intervention review of progress against goals and further goals to be sets. In the feasibility study the NAP SACC UK Partners were Health Visitors; in the full trial Local Authorities will chose appropriate health staff.

### Quantitative analysis

Valid accelerometer data will be at least 2 days of data worn for at least 6 h per 24 h, informed by the methodology used by Pate et al. [53]. Given the variability of opening times and child attendance between nurseries, the minimum number of hours in order to be categorised as a ‘nursery day’ will be explored and a suitable cut-off used. Periods of 60-min with zero values will be interpreted as time that the monitor is not worn. A day will be considered valid if ≥ 6 h of data are recorded on a day when the child attended nursery. Children with ≥ 2 nursery days of accelerometer data will be included in the analyses. Mean minutes of sedentary time (using two thresholds of 0–25 and 0–199 counts per 15 s using the criteria proposed by Evenson and Puyau [54, 55]) will be used and mean minutes of light, moderate to vigorous intensity physical activity will be processed (thresholds of 200–799; and >  = 800 counts per 15 s) [56]. Mean accelerometer counts per minute, which provides an indication of the overall volume of physical activity in which the children engage will also be calculated as this approach facilitates comparison with studies that may have applied a different cut-point. The accelerometer data will be checked for outliers. Informed by previous studies with children we will exclude implausibly high values, such as might occur when a participant uses a trampoline, using a cap of 11,714 counts per minute (cpm) [57].

Total eating occasion size (kcal per occasion) will be computed from the sum of energy in each portion food or drink consumed for each snack (morning or afternoon) or lunch consumed in nursery. The average total size of eating occasions consumed within nursery for each child will then be derived (primary outcome). Specific foods will also be classified as core or non-core and the total intake (kcal) of core and non-core foods will be separately summed in each eating occasion consumed at nursery and expressed as a percentage of total energy consumed in an eating occasion for each child [14]. To represent the balance of healthy to less-healthy food intake consumed, the average percentage of core and non-core food in lunch and morning/afternoon snacks consumed by each child will be calculated.

The primary and secondary analyses will be pre-specified in a statistical analysis plan which will be written whilst blinded to the accumulating outcome data and will be made publicly available before the conclusion of the follow-up period. The evidence for an overall intervention effect on the primary outcomes (physical activity and nutrition) will be estimated using a multilevel linear regression model, which will include the following nursery level covariates: intervention group, IMD as used to stratify the allocation, local authority, and a random effect to accommodate variation between nurseries (clustering). The intervention effects will be presented as differences in average total activity and total energy consumed per eating occasion with their 95% confidence intervals. The exact specification of the primary analysis will be informed by an inspection of the baseline measurements of the primary outcomes.

The primary analysis approach will be adapted to estimate the intervention effect on each of the secondary outcomes, utilizing univariate multilevel linear regression (continuous outcome measures) or univariate multilevel logistic regression (binary outcome measures).

We will examine whether the intervention effect on the two primary outcome measures varies by sub-groups of participants. These sub-groups will be pre-specified in the statistical analysis plan and may include parental employment status, geographical area, child’s gender and time spent in nursery.

Sensitivity analyses will repeat the primary analysis with variations to the method that include the following (i) additional covariates where one or more measures was found to be unbalanced at baseline; (ii) missing data imputed under different assumptions about the mechanisms leading to those data being missing; and (iii) excluding outliers if inspection reveals that outliers are genuine.

### Qualitative analysis

Information collected from document analysis, questionnaires and structured elements of the training sessions/workshops observations will be entered into the REDCap data management system or an Excel file. Interview transcripts, qualitative observations and fieldnotes will be uploaded into NVivo 12 to aid data management and analysis and analysed to identify key themes. An initial coding framework will be developed by two staff including both deductive codes derived from research questions and inductive codes identified from initial readings of early transcripts. This framework will be independently applied to two to four further transcripts depending on the consistency of coding; any discrepancies in coding will be discussed and appropriate revisions made. The final framework will be applied to all subsequent transcripts with any additions or revisions recorded. We will triangulate between different process evaluation data sources (observations, questionnaires, documentary data and interviews) to identify confirmatory or contradictory results. For example, we will compare data from the observations of training workshops with the staff evaluation forms and comments from nursery manager and/or Partner interviews to understand how the workshops were received and their importance within the intervention as a whole.

### Health economic analysis

The primary economic analysis will consist of a within-trial cost consequences analysis (CCA) from the perspective of the local government, nursery and parents. Results from the within-trial CCA will allow the costs and consequences to be presented clearly in a disaggregated format rather than summarised into a single index. If there is an important difference in physical activity and/or diet at T1, a secondary analysis considering the potential longer-term costs and outcomes of the intervention will be considered. This would include a review of the economic evidence on the medium- and long-term costs and consequences of changes in physical activity and diet in young children.

## Discussion

The trial aims to evaluate the effectiveness and cost-effectiveness of the NAP SACC UK intervention to increase physical activity, reduce sedentary time and improve the quality and quantity of nutritional intake. The trial builds on the success of the feasibility study, the evaluations of the intervention and subsequent adoption across the US. The trial initially started in July 2019 and was paused from March 2020 to January 2022 because of the disruption to research in childcare settings arising from COVID-19. Following discussion with nurseries, collaborators and the funder, we restarted the study in February 2022. Some of the initial outcome measures and processes were refined and this protocol represents the study upon restarting in 2022.

If the NAP SACC UK intervention is found to be effective, this will have important policy and practice implications for the commissioning of programmes to prevent obesity, improve physical activity and nutrition in early years settings in the UK. We also have the scope to apply for additional funding to explore longer term impacts on z-BMI, height and weight beyond the early years’ settings. The trial is deliberately pragmatic in the use of public health staff or staff from commissioned services who work on physical activity and nutrition beyond health visitors as used in the feasibility trial.

## Data Availability

The Chief Investigator will manage access rights to the data set. Prospective new users must demonstrate compliance with legal, data protection and ethical guidelines before any data are released. We anticipate that anonymised trial data will be shared with other researchers to enable meta-analyses.

## Abbreviations

BMI: Body Max Index
CCA: Cost consequence analysis
CI: Confidence Interval
COVID-19: Coronavirus disease
CPM: Counts per Minute
CTU: Clinical Trials Unit
IMD: Index of Multiple Deprivation
MVPA: Moderate to Vigorous Physical Activity
NDNS: National Diet and Nutrition Survey
NHS: National Health Service
NIHR: National Institute for Health Research
NIHR PHR: NIHR Public Health Research programme
PA: Physical Activity
PBRC: Pennington Biomedical Research Centre
PedsQL: Pediatric Quality of Live Inventory
RCT: Randomised Control Trial
REC: Research Ethics Committee
REDCap: Research Electronic Data Capture
RFPM: Remote Food Photography Method
SCT: Social Cognitive Theory
SD: Standard Deviation
SEDN: Special Educational Needs and Disabilities
TIDieR: Template for Intervention Description and Replication
TSC: Trial Steering Committee
UK: United Kingdom
USA/US: United States of America/United States
UOB: University of Bristol
zBMI: BMI Z-score
